# Multiple mechanisms of theta rhythm generation in a model of the hippocampus

**DOI:** 10.1186/1471-2202-16-S1-O17

**Published:** 2015-12-18

**Authors:** Ali Hummos, Satish S Nair

**Affiliations:** 1 Department of Health Informatics, University of Missouri, Columbia, MO 65211, USA; 2 Department of Psychiatry, University of Missouri, Columbia, MO 65211, USA; 3 Department of Electrical & Computer Engineering, University of Missouri, Columbia, MO 65211, USA

## 

Hippocampal theta oscillations (4-12 Hz) are consistently recorded during memory tasks and spatial navigation. While computational models suggested specific mechanisms for theta generation, experimental inactivation of these mechanisms did not disrupt theta, precluding definitive conclusions about their roles. We investigated this discrepancy using a biophysical model of the hippocampus that included several of the components implicated in rhythm generation, all constrained by prior experimental results. The CA3 network model included recurrently connected pyramidal cells, and inhibitory basket cells (BC) and oriens-lacunosum moleculare (OLM) cells. The model was developed by matching experimental results characterizing neuronal firing patterns, synaptic dynamics, short-term synaptic plasticity and the three-dimensional organization of the hippocampus. The model revealed four mechanisms that generated theta oscillations: intrinsic theta resonance of pyramidal cells, recurrent connections between them, coupling between OLM and pyramidal cells, and, as a novel finding, the correlated input from entorhinal cortex. Consistent with experimental results, inactivation of any single mechanism did not disrupt the rhythm. Another novel finding was that the low and high cholinergic states differentially recruited theta generating mechanisms. Atropine -sensitive and -resistant forms of theta, however, corresponded to theta generated during low and high levels of network excitation, respectively. These findings provided an alternative interpretation of the atropine-based classification of theta oscillations, and suggested that the theta rhythm is an intrinsic property of the network. Any experimental manipulation or brain state that enhances or suppresses excitation might also, therefore, non-specifically enhance or suppress theta oscillations.

